# Cross-species interference of gene expression

**DOI:** 10.1038/s41467-018-07353-0

**Published:** 2018-11-27

**Authors:** Irene de Bruijn, Koen J. F. Verhoeven

**Affiliations:** 1 Department of Microbial Ecology, Netherlands Institute of Ecology (NIOO-KNAW), Droevendaalsesteeg 10, Wageningen, 6708PB The Netherlands; 2 Department of Terrestrial Ecology, Netherlands Institute of Ecology (NIOO-KNAW), Droevendaalsesteeg 10, Wageningen, 6708PB The Netherlands

## Abstract

Microbes can contribute to protection of animals and plants against diseases. A recent study reveals a mechanism by which a bacterium controls fungal infection in wheat, involving secretion of a metabolite that affects histone acetyltransferase activity of a plant pathogenic fungus.

## Introduction

In plant microbiomes, different microbes interact and compete with each other for nutrients and living space. Some of these microbes are beneficial to the plant by inhibiting plant pathogens via, for example, the production of antimicrobial metabolites such as phenazines.

Phenazines are pigments produced by a wide variety of microorganisms, including *Pseudomonas* bacteria, and can inhibit the growth of fungal and nematode plant pathogens. In addition, some phenazines, such as pyocyanin in the human pathogen *Pseudomonas aeruginosa*, play roles as virulence factors^1^. Phenazines can accept or donate electrons, and therefore one of their major modes of action is the generation of reactive oxygen species (ROS), inducing an oxidative stress response that can potentially lead to cell lysis^1^.

Now, Chen et al.^2^ report on a different mechanism by which a phenazine inhibits the growth and virulence of *Fusarium graminearum*, a fungus that causes disease on wheat. They show that a phenazine produced by the bacterium *Pseudomonas piscium*, a member of the wheat microbiome, inhibits the activity of a fungal acetyltransferase thus reducing the acetylation of specific histones in the fungus. As histone acetylation is an important epigenetic mechanism involved in regulation of gene expression, the results indicate that the phenazine may be affecting gene regulation in the fungal pathogen, resulting in strongly reduced virulence. This work thus points at cross-species manipulation of gene expression as, potentially, an underappreciated mechanism underlying the action of biological control agents.

### The epigenetic machinery

In eukaryotes (such as animals, plants, and fungi), epigenetic mechanisms include DNA cytosine methylation and histone modifications. Eukaryotic DNA is highly compacted and wrapped around histone proteins. Post-translational modifications of histone tails, such as methylation-demethylation and acetylation–deacetylation, affect chromatin structure and can activate and deactivate gene expression^3^. In bacteria, which do not have histones, DNA adenine and cytosine methylation is the major epigenetic modification that influences the transcription of genes and that also protects DNA from cleavage by bacterial restriction-modification systems (which attack foreign DNA)^3^.

### Manipulation of epigenetic machinery in cross-species interactions

In antagonistic species interactions, compromising the gene regulatory epigenetic machinery of your opponent can be a powerful weapon. This is frequently observed in host-pathogen interactions, where the pathogen manipulates host gene expression to evade the immune response. For example, the human pathogen *Listeria monocytogenes* produces toxins that affect histone deacetylation in host cells, resulting in reduced expression of immune response genes (reviewed in Grabiec and Potempa^4^). Two other bacterial pathogens—*Mycobacterium tuberculosis* and *Helicobacter pylori*—can also decrease histone acetylation in host cells^4^, for instance by activating repressors of histone deacetylase in *M*. *tuberculosis* infections. Furthermore, an effector protein produced by the plant pathogenic oomycete *Phytophthora sojae* suppresses histone acetylation levels at the promoters of multiple defence-related genes by interfering in the assembly of histone acetylation complexes, thereby increasing plant susceptibility to infection^4^.

An intriguing observation is that the genomes of many pathogenic bacteria, which lack typical histones and chromatin, include genes for proteins containing SET domains, which in eukaryotes are involved in post-translational modification of histones^5^. This could indicate that manipulation of host epigenetic machinery by bacterial pathogens might be more common than previously thought. Indeed, *Chlamydia* bacteria secrete SET-domain proteins during infection which associate with host cell nuclei and target the eukaryotic histones^6^.

Beyond host-pathogen interactions, microbes can induce changes in the activity of the epigenetic machinery in other types of interactions. For example, the recent study by Chen et al.^2^ shows that a plant beneficial bacteria secretes phenazine that inhibits histone acetyltransferase function, production of virulence factors and expression of genes involved in host cell penetration and plant cell wall degradation in *Fusarium graminearum*. Similarly, the soil-dwelling bacterium *Streptomyces rapamycinicus* increases histone acetylation in the fungus *Aspergillus nidulans* and increases the production of secondary metabolites like penicillin and orsellinic acid^7^.

Modulation of the epigenetic machinery has also been observed in interactions between plants. For example, wheat produces a precursor of a histone deacetylase inhibitor, which is released by the plant into the soil, broken down (possibly by soil microbes) into an active form, and then taken up by roots of neighboring plants where histone deacetylase activity is compromised and growth is reduced^8^.

Not only antagonistic, but also symbiotic interactions may be established via cross-species manipulation of the epigenetic machinery. For instance, and although details of the mechanism remain to be discovered, infection of insects by *Wolbachia* bacteria triggers upregulation of a host DNA methyltransferase, which subsequently determines resistance of the insect to an RNA virus^9^.

Taken together, these studies suggest that cross-species manipulation of epigenetic function may be a widespread phenomenon, with a broad range of possible effects on the outcome of the interactions.

### Interference via small RNAs

Cross-species manipulation can also occur via exchange of small RNAs (sRNAs). The potential of sRNAs for regulating genes in a sequence-specific way with high precision makes them suitable regulators of gene expression not only within an organism but also between organisms, and sRNA trafficking has been suggested as a new way of communication between different interacting species^10^. For both eukaryotes and prokaryotes, the trafficking of sRNAs across species and kingdoms mostly occurs via extracellular vesicles to avoid the degradation by RNases in the extracellular environment^11^. For example, a bidirectional exchange of sRNAs via extracellular vesicles has been observed between the fungal plant pathogen *Botrytis cinerea* and *Arabidopsis*, by which the fungal pathogen suppresses the plant immune response^12^ and the plant suppresses virulence of the pathogen^13^. Also the bacterial pathogen *Pseudomonas aeruginosa* can attenuate the human immune response by secreting outer membrane vesicles containing a specific sRNA that targets MAP-kinase mRNAs in the host’s signaling pathway to the immune response^14^.

Therefore, the exchange of sRNAs may be common in inter-species interactions, but this is mostly studied in pathogenic, parasitic or symbiotic interactions and far less for commensal or beneficial bacteria. It is possible that sRNAs may play roles in the colonization of animal and plant hosts by commensal microbes, perhaps helping the host to distinguish friends from foes.

## Conclusions

Cross-species manipulation of gene expression clearly plays an important role in the interactions between organisms but the underlying mechanisms are understudied, in comparison with those by which organisms regulate the expression of their own genome. Observations thus far show an intriguing array of cross-species manipulations in a broad range of interaction types. While much work has emphasized host-pathogen interactions, it will be important to better understand the mechanisms underlying other interactions, such as those involved in biocontrol activity of plant beneficial microbes or in the interaction between commensal microbes and their hosts.

It is tempting to speculate that the mechanisms behind cross-species interactions could one day be applied for the development of more sustainable methods for crop protection, especially given the rise of fungicide resistant plant pathogenic fungi. For instance, can plant leaves be sprayed with specific small RNAs targeting pathogenicity genes as a more sustainable method to control plant pathogens^15^? Or can naturally occurring beneficial microbes be stimulated to increase the production of antimicrobial metabolites, such as phenazine, thereby controlling crop pathogens? The ways in which interacting species affect each other’s gene expression, and possibly develop resistance against manipulation by other species, is a largely unexplored research area. A better understanding of these mechanisms will provide a new dimension for fundamental and applied research to combat diseases.
